# Ruxolitinib Modulates P-Glycoprotein Function, Delays T Cell Activation, and Impairs CCL19 Chemokine-Directed Migration in Human Cytotoxic T Lymphocytes

**DOI:** 10.3390/ijms26136123

**Published:** 2025-06-26

**Authors:** Kipchumba Biwott, Algirmaa Lkhamkhuu, Nimrah Ghaffar, Albert Bálint Papp, Nastaran Tarban, Katalin Goda, Zsolt Bacso

**Affiliations:** 1 Department of Biophysics and Cell Biology, Faculty of Medicine, University of Debrecen, 4032 Debrecen, Hungary; kipchumba.biwott@med.unideb.hu (K.B.); lkhamkhuu.algirmaa@med.unideb.hu (A.L.); ghaffar.nimrah@med.unideb.hu (N.G.); goda@med.unideb.hu (K.G.); 2 Doctoral School of Molecular Cell and Immune Biology, Faculty of Medicine, University of Debrecen, 4032 Debrecen, Hungary; nastarantarban88@gmail.com; 3 Department of Biological and Life Sciences, Technical University of Kenya, Nairobi 52428-00200, Kenya; 4 Department of Biomedicine and Pharmacy, Darkhan-Uul Medical School, Mongolian National University of Medical Sciences, Ulan Bator 14210, Mongolia; 5 Department of Biochemistry and Molecular Biology, Faculty of Medicine, University of Debrecen, 4032 Debrecen, Hungary; papp.albert@dental.unideb.hu; 6 Doctoral School of Dental Sciences, Faculty of Medicine, University of Debrecen, 4032 Debrecen, Hungary; 7 Dean’s Office, Faculty of Pharmacy, University of Debrecen, 4032 Debrecen, Hungary

**Keywords:** ruxolitinib, Cytotoxic T lymphocytes (CTLs), P-glycoprotein (Pgp; MDR1; ABCB1), T cell activation, CCL19 chemokine-driven migration, JAK-STAT signaling, immune modulation

## Abstract

Ruxolitinib, a clinically approved JAK1/2 inhibitor used in the treatment of hematologic malignancies and inflammatory conditions, has been shown to interfere with the function of cytotoxic T lymphocytes (CTLs). Previous studies supported the involvement of the multidrug resistance transporter P-glycoprotein (Pgp/ABCB1) in CTL biology; however, the nature of its regulation remains unclear. To address this, we investigated the impact of ruxolitinib on Pgp expression and function in human CD8^+^ T cells. We demonstrate that CD8^+^ T lymphocytes express Pgp dynamically at both the mRNA and protein levels across naïve, short-term, and long-term activation states. Ruxolitinib increased the calcein accumulation in human Pgp-overexpressing NIH-3T3 cells and in CTLs and directly modulated Pgp function by increasing its basal ATPase activity in a concentration-dependent manner (10–100 μM), similar to the effect of the known Pgp substrate/modulator verapamil. Although measurable ATPase stimulation and transport inhibition were observed at supratherapeutic concentrations of ruxolitinib, its Pgp-mediated efflux may also occur at therapeutically relevant concentrations. In contrast, at therapeutically relevant plasma concentrations (1–3 μM), ruxolitinib significantly stabilized the mRNA expression of Pgp during early T-cell receptor (TCR) activation and inhibited the TCR-induced upregulation of Pgp, CD8, and PD-1 surface markers, suggesting its interference with activation-associated differentiation. At these same concentrations, ruxolitinib also impaired CCL19-directed transmigration of CTLs across human umbilical vein endothelial cell (HUVEC) monolayers, indicating disruption of lymphoid homing cues. Collectively, these findings demonstrate that ruxolitinib modulates Pgp at both the transcriptional and functional levels, with distinct concentration dependence. The ability of ruxolitinib to alter CTL activation and migration at clinically relevant plasma concentrations highlights the need for careful evaluation of JAK inhibitor–mediated immunomodulation and its implications for vaccination, transplantation, and T cell-based immunotherapies.

## 1. Introduction

P-glycoprotein (Pgp, also known as MDR1 and encoded by the *ABCB1* gene) is a critical member of the ATP-binding cassette (ABC) family of membrane transporters. It functions as an efflux pump, transporting a wide range of xenobiotics, toxins, and cytostatic drugs out of cells [1,2,3,4]. Pgp is widely expressed in protective barriers such as the blood-brain barrier, gastrointestinal tract, liver, kidneys, and placental tissues. Notably, Pgp is expressed in immune cells, including natural killer (NK) cells, dendritic cells (DCs), antigen-presenting cells, and both CD4^+^ and CD8^+^ T lymphocytes [5,6,7,8].

In CD8^+^ T cells, Pgp plays key roles in mediating multidrug resistance, regulating immune responses, protecting against reactive oxygen species generated during early T-cell receptor (TCR) activation, and facilitating T-cell activation and expansion [6,9,10]. Beyond its role in drug efflux, Pgp also contributes to the release of pro-inflammatory cytokines and cholesterol, with broad implications for immune function and drug bioavailability [11].

The impact of Pgp-mediated drug resistance is well documented in oncology [12], particularly regarding tyrosine kinase inhibitors (TKIs) such as imatinib in chronic myeloid leukemia (CML) or others in [13]. In myeloproliferative neoplasms (MPNs), disorders frequently associated with JAK-STAT pathway mutations, Pgp similarly contributes to drug resistance [14]. While JAK1 and JAK2 proteins are essential for cytokine-mediated signaling in normal hematopoietic cells, their dysregulation promotes oncogenesis in conditions such as polycythemia vera, essential thrombocythemia, primary myelofibrosis, and acute lymphoblastic leukemia [15,16]. These insights led to the development of JAK inhibitors, notably ruxolitinib, a clinically approved non-selective JAK1/2 inhibitor for the treatment of myelofibrosis and other MPNs [17,18,19,20,21]. Ruxolitinib is also increasingly used for immunomodulation in conditions like graft-versus-host disease (GVHD) [18,22] or even with other new indications [23,24,25].

Ruxolitinib’s pharmacokinetics are primarily influenced by the cytochrome P450 enzyme CYP3A4, with lesser influence by CYP2C9, and are known to have interactions that affect its metabolism and efficacy [26,27,28]. In addition to CYP-mediated metabolism, ruxolitinib’s interaction with Pgp could significantly impact its distribution, bioavailability, and therapeutic activity, particularly in patients receiving combination therapies involving other Pgp substrates. Interestingly, the absorption of ruxolitinib is not significantly affected by P-glycoprotein in the gut [29]. However, genetic inactivation of Pgp has been shown to enhance ruxolitinib’s inhibition of T cell proliferation, suggesting that Pgp-mediated efflux may attenuate its immunosuppressive effects [22].

Programmed cell death-1 (PD-1, CD279), a member of the CD28 family, is an inhibitory receptor expressed at low levels on resting T and B cells. Upon TCR or BCR activation, PD-1 is upregulated to regulate immune tolerance and prevent autoimmunity [30,31]. Various cancers exploit PD-1 signaling to evade immune detection, and ruxolitinib has been shown to modulate PD-1 pathways, enhancing anti-tumor immune responses [32]. However, while Pgp’s role as an efflux transporter is well established, its interaction with ruxolitinib remains poorly understood or even controversial [29,33]. It is unclear whether ruxolitinib acts as a substrate, modulator, inhibitor, or inducer of Pgp [11,22,34,35].

In this study, we investigated the dynamic regulation of Pgp and CD8 expression in human cytotoxic T lymphocytes under resting and antigen-primed conditions. We assessed the functional interaction between ruxolitinib and Pgp by applying a substrate accumulation assay and measuring ATPase activity. Our results demonstrate that ruxolitinib increases calcein accumulation by Pgp-expressing cells and elevates the ATPase activity of Pgp. Although the above effects were observed at concentrations higher than the typical therapeutic plasma levels of ruxolitinib, they reflect a direct molecular interaction between ruxolitinib and Pgp. Additionally, ruxolitinib delays the downregulation of *ABCB1* mRNA during T-cell activation, suppresses PD-1 upregulation, and significantly impairs CCL19-directed CTL transmigration across human endothelial barriers. These findings suggest that ruxolitinib modulates T cell differentiation, trafficking, and efflux capacity, with important implications for immune modulation and therapeutic strategies.

## 2. Results

To systematically investigate the influence of ruxolitinib on cytotoxic T lymphocyte (CTL) phenotype and function, we carried out a series of molecular, biochemical, and cellular assays. First, we characterized baseline and antigen-induced changes in P-glycoprotein and CD8 expression, then evaluated ruxolitinib’s effects on Pgp mRNA levels, transporter activity, activation marker expression, and chemotactic behavior.

### 2.1. Dynamic Expression of P-Glycoprotein and CD8 in Quiescent and Antigen-Primed Human Lymphocytes

Pgp plays a critical role in mediating drug efflux and regulating immune cell function. Its expression is dynamically modulated during T cell activation and differentiation. To investigate changes in Pgp expression, human peripheral blood lymphocytes were co-cultured with proliferation-inhibited JY cells in a mixed lymphocyte culture assay. The JY cell line, derived from human B-lymphoblasts, constitutively expresses high levels of the MHC class I molecule A* 02:01 on its surface. This makes it highly suitable as an antigen-presenting cell for stimulating T cell responses [36,37].

**Figure 1:**
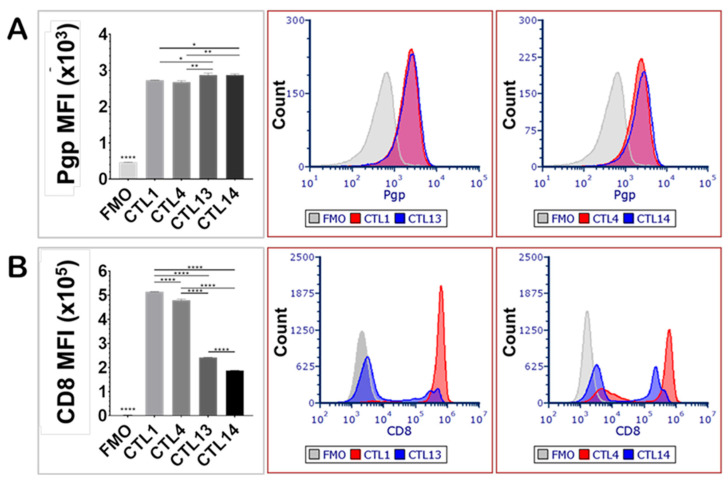
Expression of P-glycoprotein and CD8 on JY-primed and unprimed human CD8^+^ T lymphocytes. Pgp expression decreased, while CD8 expression increased upon activation. (**A**) Mean fluorescence intensity (MFI) and fluorescence intensity distribution overlays of Pgp in JY-primed (CTL1, CTL4) and unprimed (CTL13, CTL14) cytotoxic T lymphocyte (CTL) samples from different human donors. (**B**) MFI and fluorescence distribution overlays of CD8 expression in CTL1, CTL4, CTL13, and CTL14 donor samples. Statistical comparisons between groups were performed using one-way ANOVA followed by Tukey’s multiple comparison test (n = 3). Significance levels are indicated as * *p* < 0.05, ** *p* < 0.01, **** *p* < 0.0001. Error bars represent mean ± SEM.

P-glycoprotein expression was evaluated in quiescent peripheral blood CD8^+^ lymphocytes (CTL13, CTL14) and compared with CD8^+^ lymphocytes primed with JY cells (CTL1, CTL4), with each blood sample derived from a different human donor (Figure 1). The priming procedure involved culturing peripheral blood lymphocytes with JY cells biweekly over a period of one month, thereby facilitating the differentiation of mature cytotoxic T lymphocytes and memory T cells. Unprimed lymphocytes (CTL13, CTL14) exhibited slightly but significantly higher levels of Pgp expression compared to the JY-primed lymphocytes (CTL1, CTL4) (Figure 1A). This modest decrease in average Pgp expression observed after repeated antigenic stimulation likely reflects the pronounced transition of T cells from a naïve to an effector phenotype, accompanied by the generation of memory cells, each population demonstrating distinct patterns of Pgp expression.

In contrast, CD8 expression was significantly elevated in JY-primed CTLs (CTL1, CTL4) compared to their unprimed counterparts (CTL13, CTL14) (Figure 1B). This increase in CD8 expression likely reflects enhanced T cell activation, differentiation, and cytotoxic potential resulting from repeated antigen stimulation, thereby promoting robust CD8^+^ cellular immune responses in the mixed lymphocyte cultures. Notably, CD8 expression levels showed considerable variation among individual donors, underscoring donor-dependent heterogeneity in protein expression. In contrast, inter-donor variability in Pgp expression was not statistically significant.

### 2.2. Ruxolitinib Modulates P-Glycoprotein mRNA Expression in TCR-Activated Human T Cells

*ABCB1*, the gene encoding P-glycoprotein, plays a pivotal role in chemoresistance and immune cell function by mediating the efflux of a wide range of xenobiotics. To investigate the potential interaction between the Janus kinase (JAK) inhibitor ruxolitinib and Pgp, we assessed its effects on *ABCB1* mRNA expression in primary human T lymphocytes.

**Figure 2:**
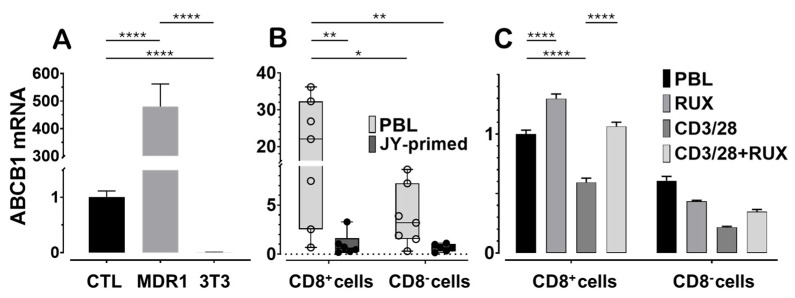
Human P-glycoprotein transporter (*ABCB1*) mRNA is expressed in cytotoxic T lymphocytes. Gene expression levels were normalized to ACTB (β-actin). (**A**) *ABCB1* mRNA expression in JY-primed cultured human cytotoxic T lymphocytes compared to control cell lines: *ABCB1*-transfected (MDR1, expressing human ABCB1) and untransfected NIH-3T3 mouse fibroblasts. Values are presented as fold changes relative to the average mRNA expression in CTLs. (**B**) *ABCB1* mRNA levels in flow cytometry-sorted CD8^+^ and CD8^−^ T cells from freshly isolated peripheral blood lymphocytes (PBLs) and JY-primed CTLs cultured for one month from the same donors. Values are expressed as fold changes relative to the average expression in JY-primed CD8^+^ T cells. (**C**) *ABCB1* mRNA expression in flow cytometry-sorted CD8^+^ and CD8^−^ T cells from freshly isolated PBLs were activated or not for 72 h with anti-CD3/CD28 beads, either in the presence or absence of 100 nM ruxolitinib (RUX), a JAK1/2 kinase inhibitor. Values are shown as fold changes relative to the average expression in unstimulated PBLs. Statistical comparisons were performed using one- or two-way ANOVA, followed by Dunnett’s or Tukey’s multiple comparisons tests, as appropriate. Significance levels are indicated by asterisks (* *p* < 0.05; ** *p* < 0.01; **** *p* < 0.0001). Error bars represent the mean ± SEM (n = 3) in panels A and C. In panel B, the median, interquartile range (second and third quartiles), and 95% confidence intervals are shown (n = 6–7).

We first assessed *ABCB1* mRNA expression in JY-primed lymphocyte cultures, which are predominantly composed of CD8^+^ cytotoxic T lymphocytes, and compared these levels to a positive control cell line known to highly express human Pgp. The NIH-3T3 MDR1 cell line, mouse fibroblasts transfected with the human *ABCB1* gene, served as the positive control, while untransfected NIH-3T3 parental cells served as the negative control (Figure 2A). When normalized to CTL expression levels, NIH-3T3 MDR1 cells expressed approximately 500-fold higher levels of *ABCB1* mRNA, whereas expression in the untransfected NIH-3T3 cells was undetectable. Gene expression normalization was performed using both human and mouse ACTB (β-actin) and GAPDH reference genes. For consistency, all figures display normalization to ACTB, which yielded lower inter-treatment variability in human lymphocytes compared to GAPDH.

Next, we examined baseline variability in *ABCB1* mRNA levels between CD8^+^ T cells isolated from peripheral blood lymphocytes and those from JY-primed CTL cultures generated from five independent human donors (Figure 2B). JY cells provided alloantigenic stimulation, mimicking physiological T cell priming. After this priming, *ABCB1* mRNA levels were reduced by approximately 95%, or to one-twentieth of those observed in naïve PBL-derived CD8^+^ cells. Although substantial donor-to-donor variation was observed in the naïve cell populations, a consistent decrease in *ABCB1* expression was noted across nearly all donors after long-term activation. This trend parallels our earlier findings at the protein level and is consistent with T cell maturation, in which cells shift from naïve and memory states toward more differentiated effector phenotypes [37].

Finally, we investigated the immediate impact of ruxolitinib on *ABCB1* expression in CD8^+^ T lymphocytes undergoing acute T cell receptor (TCR) stimulation (Figure 2C). PBLs were stimulated for 72 h using anti-CD3/CD28 beads, which led to a reduction in *ABCB1* mRNA expression, consistent with the trend observed in the one-month JY-priming model. Notably, co-treatment with 100 nM ruxolitinib attenuated this decrease and appeared to sustain *ABCB1* expression in both CD8^+^ and CD8^−^ lymphocyte populations. These findings suggest that ruxolitinib may modulate Pgp expression by interfering with TCR signaling pathways involved in T-cell activation and differentiation.

In our previous experiments, we observed similar trends at the protein level of Pgp [37], whereas our current experiments demonstrated these trends at the mRNA level.

### 2.3. Dose-Dependent Inhibition of P-Glycoprotein Activity by Ruxolitinib and Zosuquidar in Cytotoxic T Lymphocytes

To evaluate the presence of functional P-glycoprotein in human cytotoxic T lymphocytes, we monitored its transporter activity using a calcein efflux assay [38,39,40]. Calcein-AM is a fluorescent, nonpolar substrate of Pgp that becomes fluorescent upon intracellular esterase cleavage. Its intracellular accumulation is inversely proportional to Pgp efflux activity. To quantify transporter function, we calculated the transport activity factor (TAF), representing the fractional increase in calcein accumulation in the presence versus absence of a Pgp inhibitor.

**Figure 3:**
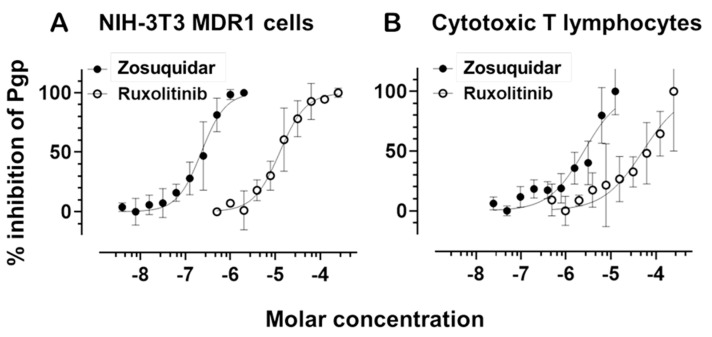
Dose-dependent inhibition of P-glycoprotein activity by ruxolitinib and zosuquidar. (**A**) NIH-3T3 cells stably expressing human MDR1 and (**B**) primary human cytotoxic T lymphocytes were analyzed using a calcein-AM efflux assay to assess Pgp activity. Cells were treated with increasing concentrations of either zosuquidar (filled circles) or ruxolitinib (open circles), and intracellular fluorescence was measured to determine the percentage inhibition of Pgp. Data represent mean ± SD from triplicate measurements. Zosuquidar showed potent inhibition in both cell types, with lower IC_50_ values in NIH-3T3 MDR1 cells compared to CTLs. Ruxolitinib inhibited Pgp with significantly higher IC_50_ values, indicating lower potency in both cell types.

To validate Pgp activity and evaluate its pharmacological inhibition, we generated dose-response curves using the third-generation Pgp-specific inhibitor zosuquidar. These curves were established in both NIH-3T3 cells overexpressing human MDR1 (NIH-3T3 MDR1) and in primary human CTLs. In both systems, zosuquidar effectively inhibited Pgp-mediated efflux in a dose-dependent manner (Figure 3). NIH-3T3 MDR1 cells exhibited a high sensitivity to zosuquidar, with an IC_50_ range of 1.779 × 10^−7^ to 2.847 × 10^−7^ M. In contrast, CTLs displayed a right-shifted inhibition curve, with an IC_50_ range of 1.809 × 10^−6^ to 3.498 × 10^−6^ M, consistent with their lower baseline Pgp expression in these cells.

To optimize assay sensitivity in CTLs, we reduced the concentration of calcein-AM to account for their lower transporter expression relative to NIH-3T3 MDR1 cells. This adjustment enabled a clear and quantifiable dose-dependent inhibition of Pgp, confirming that zosuquidar remains effective in primary CTLs, albeit at higher concentrations than in Pgp-overexpressing cells.

Next, we evaluated the JAK1/2 inhibitor ruxolitinib for its potential to modulate Pgp function. Ruxolitinib also produced a measurable, dose-dependent inhibition of calcein-AM efflux in both NIH-3T3 MDR1 cells and CTLs. In NIH-3T3 MDR1 cells, the IC_50_ for ruxolitinib ranged from 1.002 × 10^−5^ to 1.572 × 10^−5^ M, indicating moderate inhibitory potency. In CTLs, the IC_50_ was higher, between 3.003 × 10^−5^ and 8.543 × 10^−5^ M, suggesting reduced affinity because of the lower Pgp expression compared to the NIH-3T3 MDR1 cells. The dose-response curves for ruxolitinib were substantially right-shifted in both systems compared to zosuquidar, suggesting a weaker interaction with the transporter. Nevertheless, the inhibition profile in CTLs suggests that ruxolitinib may act as a low-affinity Pgp inhibitor or modulator.

### 2.4. Ruxolitinib Directly Stimulates P-Glycoprotein ATPase Activity and Interferes with Verapamil-Induced Activation

To evaluate the direct interaction of ruxolitinib with Pgp, we performed ATPase activity assays using membrane preparations from NIH-3T3 MDR1 cells. Although our fluorescent substrate accumulation assay suggested functional inhibition of Pgp, the ATPase assay provides a more direct readout of transporter interaction. Changes in ATP hydrolysis reflect alterations in the conformational cycling triggered by substrate or inhibitor binding.

**Figure 4:**
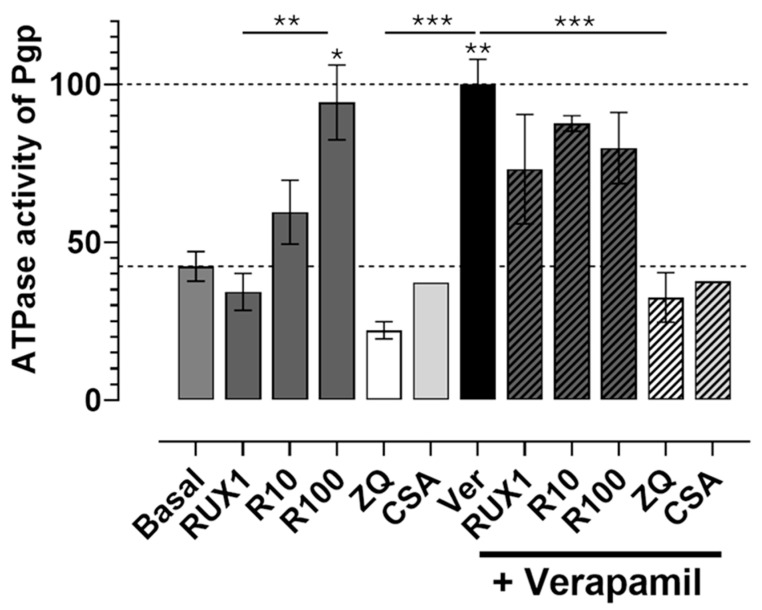
Ruxolitinib activates the ATPase activity of P-glycoprotein and interferes with other activators. Ruxolitinib (RUX) increased basal ATPase activity (lower dashed line) in a dose-dependent manner at concentrations of 1 μM (RUX1), 10 μM (R10), and 100 μM (R100), with R100 significantly enhancing basal ATPase activity to levels comparable to those induced by verapamil (Ver). Zosuquidar (ZQ) and cyclosporin A (CSA) reduced both basal and verapamil-stimulated ATPase activity (taken as 100% and indicated by the upper dashed line). Co-treatment with ruxolitinib and verapamil exhibited additive or competitive effects, depending on the concentration of ruxolitinib. Data represent mean ± SEM; significance determined by one-way ANOVA followed by post-hoc multiple comparisons: * *p* < 0.05; ** *p* < 0.01; *** *p* < 0.001 (n = 3, except CSA n = 1).

We first assessed basal ATPase activity and examined whether ruxolitinib, zosuquidar (ZQ), or cyclosporine A (CSA) could modulate it. As shown in Figure 4, ruxolitinib stimulated basal Pgp ATPase activity in a concentration-dependent manner with doses tested at 1 μM, 10 μM, and 100 μM. The highest dose (100 μM) more than doubled basal ATPase activity compared to control conditions, with the increase being statistically significant (*p* < 0.01). The effect of 100 μM ruxolitinib was comparable to that of verapamil (40 μM), a well-characterized Pgp activator, which also significantly elevated ATPase activity (*p* < 0.001).

In contrast, both ZQ (10 μM) and CSA (20 μM), known Pgp inhibitors, significantly reduced the basal ATPase activity compared to the control (*p* < 0.01 for ZQ), confirming their inhibitory effect on transporter function.

Next, we investigated how ruxolitinib modulates verapamil-induced ATPase stimulation. Co-treatment with verapamil and increasing concentrations of ruxolitinib revealed concentration-dependent interference: at lower ruxolitinib concentrations (1–10 μM), ATPase activation appeared additive to verapamil’s effect. However, at 100 μM ruxolitinib, ATPase activity was significantly reduced compared to verapamil alone (*p* < 0.001), suggesting a competitive or inhibitory interaction at higher drug levels.

Similarly, ZQ and CSA were effective in completely suppressing verapamil-induced ATPase stimulation (*p* < 0.001 compared to verapamil alone), consistent with their potent inhibitory action.

Together, these results demonstrate that ruxolitinib directly stimulates basal Pgp ATPase activity in a concentration-dependent and statistically significant manner and can interfere with ATPase activation by other substrates, such as verapamil. This observation strongly supports the interpretation that ruxolitinib acts as a direct Pgp-interacting compound, likely behaving as a transported substrate or modulator, with potential implications for drug-drug interactions involving Pgp.

### 2.5. Ruxolitinib Impedes PD-1 Expression and Modulates CD8 and Pgp Levels in Human CD8^+^ T Cells Following Acute Activation

Ruxolitinib is known to delay lymphocyte maturation, a property leveraged clinically in graft-versus-host disease and myeloproliferative disorders where altered JAK-STAT signaling drives aberrant T cell proliferation. To study whether ruxolitinib’s maturation-delaying effect could be recapitulated in vitro, we activated human peripheral blood lymphocytes with CD3/CD28 beads for 72 h. Then, we assessed the cell surface expression of PD-1, CD8, and Pgp.

Given our focus on CD8^+^ cytotoxic T lymphocytes, we analyzed the CD8^+^ and CD8^−^ subsets separately by flow cytometry. Cells were stained with fluorophore-conjugated antibodies against CD8, PD-1, and Pgp, and mean fluorescence intensity (MFI) was quantified.

**Figure 5:**
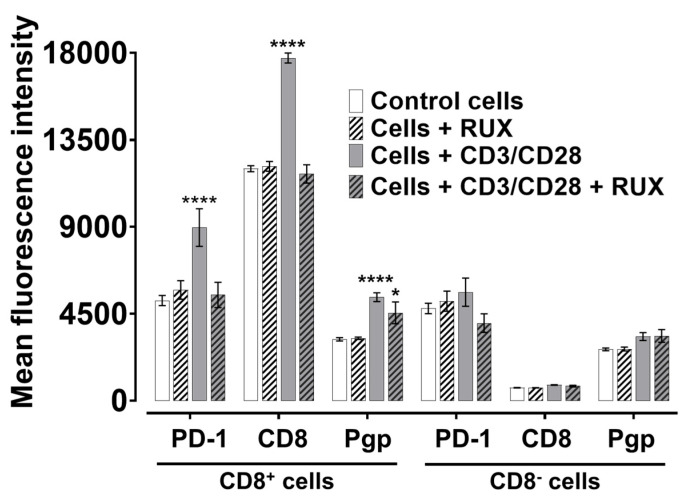
Ruxolitinib inhibits PD-1, CD8, and Pgp surface levels on sorted human cytotoxic T lymphocytes during acute CD3/CD28-mediated activation. Peripheral blood mononuclear cells were activated with CD3/CD28 beads for 72 h and treated either with ruxolitinib (100 nM) or left untreated. Following incubation, cells were stained with fluorophore-conjugated anti-PD-1, anti-CD8, and anti-Pgp antibodies and analyzed by flow cytometry. Fluorescence intensity was quantified in 20,000 cells per condition. Data represent mean ± SEM from three independent experiments (n = 3). Group comparisons were analyzed by one-way ANOVA followed by Tukey’s multiple comparison test (* *p* < 0.05; **** *p* < 0.0001).

As shown in Figure 5, acute CD3/CD28 activation robustly upregulated PD-1 expression in CD8^+^ cells (*p* < 0.0001), confirming successful T cell activation. Ruxolitinib treatment significantly blunted this induction, resulting in markedly lower PD-1 levels than activated, untreated controls (*p* < 0.0001), suggesting that ruxolitinib delays or attenuates activation-associated phenotypic maturation.

Similarly, CD8 expression levels were significantly increased upon CD3/CD28 stimulation (*p* < 0.0001). However, this upregulation was partially inhibited by ruxolitinib (*p* < 0.0001), further supporting an inhibitory effect on activation-induced changes. For Pgp, CD3/CD28 activation also significantly upregulated surface expression (*p* < 0.0001), but ruxolitinib treatment significantly suppressed this induction, although to a lesser extent than for PD-1.

In contrast, the CD8^−^ population exhibited minimal changes in PD-1, CD8, or Pgp expression across conditions, and the differences were not statistically significant. This suggests that ruxolitinib’s effects are more pronounced in CD8^+^ T cells.

Overall, these findings demonstrate that ruxolitinib impedes PD-1 induction and modulates CD8 and Pgp expression in activated CD8^+^ T lymphocytes, reinforcing its role as an inhibitor of activation-associated maturation events in T cells.

### 2.6. Ruxolitinib Inhibits CCL19-Induced Transmigration of Human Cytotoxic T Lymphocytes Across Endothelial Barriers

One of the key functional properties of memory T cells is their regulated migratory capacity, which is essential for long-term survival by enabling trafficking to supportive niches such as lymph nodes and bone marrow. To assess whether ruxolitinib influences lymph node-directed migration, we evaluated the ability of human cytotoxic T lymphocytes to transmigrate across monolayers of human umbilical vein endothelial cells (HUVECs) in response to the chemokine CCL19.

**Figure 6:**
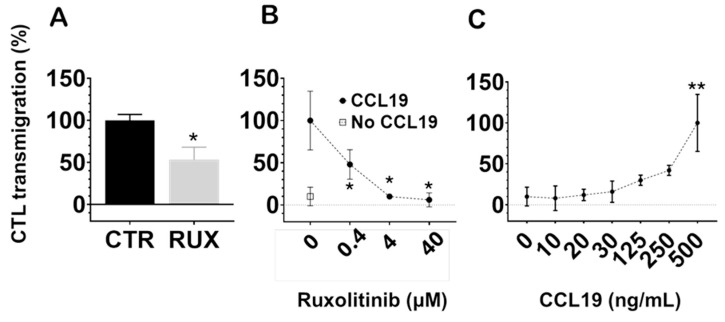
Ruxolitinib inhibits CTL transmigration induced by CCL19 through HUVEC monolayers. (**A**) Ruxolitinib (100 nM) significantly reduced CCL19-driven CTL transmigration compared to untreated control cells (CTR) (* *p* < 0.05). (**B**) Ruxolitinib inhibited CCL19-induced CTL chemotaxis in a concentration-dependent manner, with significant effects observed at 0.4 μM and above (* *p* < 0.05 compared to untreated CCL19-stimulated cells). (**C**) CCL19 induced a dose-dependent increase in CTL transmigration across HUVECs, with maximal migration observed at 500 ng/mL (** *p* < 0.01 compared to lower concentrations). Data represent mean ± SEM from at least three independent experiments (n ≥ 3). Statistical analysis was performed using one-way ANOVA followed by Tukey’s multiple comparison test.

At a pharmacologically relevant concentration (100 nM), ruxolitinib significantly inhibited CCL19-induced CTL transmigration compared to untreated controls (*p* < 0.05; Figure 6A). Notably, while suppression of Pgp efflux required higher concentrations, inhibition of CTL transmigration occurred within the therapeutic range. These findings suggest that ruxolitinib impairs the ability of CTLs to migrate across endothelial barriers in response to lymphoid chemokine signals.

Next, we examined the dose-dependence of this effect. As shown in Figure 6B, increasing concentrations of ruxolitinib progressively reduced CTL migration toward CCL19 (500 ng/mL), with significant inhibition observed at concentrations as low as 0.4 μM (*p* < 0.05), becoming more pronounced at higher concentrations. In contrast, spontaneous transmigration without CCL19 (negative control) remained minimal across all conditions.

Finally, we confirmed that CCL19 induced a robust, dose-dependent enhancement of CTL transmigration across HUVECs, as shown in Figure 6C, with maximal responses seen at the highest tested concentration (500 ng/mL, *p* < 0.01 compared to lower concentrations).

Collectively, these results demonstrate that ruxolitinib significantly impairs CTL chemotaxis across endothelial barriers in a concentration-dependent manner. This highlights a potential modulatory effect of JAK inhibition on T cell trafficking in immune environments.

## 3. Discussion

Our study provides new mechanistic insights into how ruxolitinib modulates cytotoxic T lymphocyte function through both direct and indirect effects on P-glycoprotein expression and activity, as well as the expression of T cell activation markers and chemokine-driven migration. We demonstrate that ruxolitinib not only alters Pgp transporter function at both transcriptional and protein activity levels but also impairs PD-1 upregulation and CD8 surface expression during T-cell receptor stimulation. Moreover, ruxolitinib significantly inhibits CCL19-mediated CTL transmigration across endothelial barriers, suggesting that JAK inhibition may broadly affect T cell trafficking toward lymphoid niches. These findings highlight the multifaceted impact of ruxolitinib on key processes essential for memory T cell development, immune surveillance, and potential therapeutic targeting.

### 3.1. Ruxolitinib Modulates P-Glycoprotein Expression and Activity

We hypothesized that P-glycoprotein plays a functional role in the persistence of long-lived, stem-like T lymphocytes and that their Pgp expression is dynamically regulated during T cell differentiation [37]. We observed a slight but significant decrease in Pgp protein expression in JY-primed cytotoxic T lymphocytes compared to unprimed cells. In parallel, *ABCB1* mRNA expression was approximately 20-fold higher in naïve CD8^+^ T cells than in long-term activated effector cells, supporting the notion that T cell maturation is associated with Pgp downregulation.

Excited by previous reports linking *ABCB1* expression to ruxolitinib efficacy in vitro and in vivo [22], we sought to characterize the direct interaction between ruxolitinib and Pgp.

This task appeared particularly fascinating, as earlier results [34,35] had obscured later recognized mechanisms [11], and it was assumed that ruxolitinib is not a substrate of Pgp [41]. The data used to derive those conclusions focused on transport experiments modeling gut absorption, where ruxolitinib did not indicate a harsh interaction on Pgp with other absorbing drugs [29]. However, the consequence of the ruxolitinib Pgp interaction may be more likely to occur during the extraction phase of drug redistribution when smaller concentration gradients govern more likely drug interactions for smaller molecular mass substrates [11,22,33].

Using a calcein-AM efflux assay [38,39,40], we measured inhibitory concentrations for ruxolitinib in both Pgp-overexpressing NIH-3T3 cells and primary CD8^+^ T lymphocytes. We found that ruxolitinib inhibited Pgp function with an IC_50_ of approximately 13 µM in NIH-3T3 MDR1 cells and approximately 58 µM in CD8^+^ T cells. Although these concentrations are higher than the typical 1–3 µM therapeutic plasma levels of ruxolitinib, these experiments unequivocally demonstrate that ruxolitinib interacts with Pgp. To further characterize this interaction, we performed ATPase assays using Pgp^+^ NIH-3T3 cell membrane samples. Similar to verapamil, a known substrate and modulator of Pgp, ruxolitinib induced a concentration-dependent increase in Pgp-mediated ATPase activity, achieving maximal stimulation at 100 µM. These data further confirm the direct interaction of ruxolitinib with Pgp [42,43,44,45,46]. Since classical inhibitors, such as cyclosporin A or zosuquidar, decrease the basal ATPase activity of Pgp by decreasing the turnover rate of the pump, the ruxolitinib-mediated increase in the ATPase rate reflects an increased turnover rate and most likely active transport by Pgp. Previous clinical observations suggesting that Pgp expression dampens ruxolitinib efficacy are also in line with our findings [22].

Based on Seelig’s categorization, P-gp-interacting drugs with a molecular mass lower than the critical threshold of 450 can be classified as modulators [11]. They act as substrates for Pgp, stimulate its ATPase activity, and potentially compete with other substrates. Ruxolitinib, with its molecular mass of 306.373 g/mol, may also belong to this category. Furthermore, ruxolitinib induces the mRNA expression of Pgp, a characteristic also typical of many transported compounds (see, e.g., [47,48]). Collectively, ruxolitinib has complex effects on Pgp expression and function that may impact drug-drug interactions and ruxolitinib’s bioavailability in T-cell subsets that express Pgp.

### 3.2. Effects on T Cell Activation and Phenotypic Maturation

Beyond its effects on Pgp, ruxolitinib modulated key markers of T-cell activation. During acute TCR stimulation with CD3/CD28 beads, we observed a robust upregulation of PD-1 expression, which was significantly inhibited by ruxolitinib treatment. CD8 expression and Pgp surface levels were also affected. Ruxolitinib fully or partially attenuated the upregulation of these markers following activation, respectively. Our findings align with previous reports, which show that repeated TCR activation increases PD-1 expression, whereas ruxolitinib treatment suppresses PD-1 signaling [49,50].

Interestingly, while changes in Pgp protein expression following activation were relatively modest—approximately a 50% increase after 72 h of CD3/CD28 stimulation and a slight decrease after 30 days of JY priming—mRNA levels showed markedly greater variation. Specifically, *ABCB1* mRNA expression decreased by roughly 20-fold after short-term activation and by approximately 50% after prolonged stimulation. These differences between protein and mRNA dynamics likely reflect the underlying heterogeneity of T cell subpopulations, as naïve, effector, and memory subsets differentially express Pgp. This complexity underscores the challenge of interpreting average expression levels across mixed lymphocyte populations [37]. Nonetheless, the distinct patterns of mRNA and protein expression among CTL subsets warrant further investigation to fully elucidate their regulatory mechanisms.

Together, these findings support the notion that ruxolitinib delays T-cell differentiation by interfering with activation-associated signaling pathways, thereby preserving a more naïve or less differentiated cellular phenotype.

### 3.3. Impairment of Chemokine-Directed T Cell Migration

One of the hallmarks of functional lymphocytes, particularly memory T cells, is their capacity for directed migration toward tissue niches such as lymph nodes and bone marrow [51]. Using a transmigration assay across human endothelial cell layers, we found that ruxolitinib significantly inhibited CCL19-induced chemotaxis of CTLs in a concentration-dependent manner.

This is a novel finding in human CD8^+^ T cells. Previous mouse studies suggested that ruxolitinib can impair dendritic cell and lymphocyte transmigration, possibly through both JAK1/2 inhibition and off-target effects on ROCK kinases [52,53]. In human systems, ruxolitinib has been reported to inhibit CXCL9, CXCL10, and CXCL11/CXCR3-axis-directed migration in CD4^+^ T lymphocytes and to reduce lymph node-directed migration of human dendritic cells, along with other immunomodulatory effects [50,54,55]. This study is the first to demonstrate that ruxolitinib impairs CCL19-driven migration of human CD8^+^ cytotoxic T cells.

Notably, although inhibition of Pgp efflux function and modulation of ATPase activity by ruxolitinib required concentrations higher than typical therapeutic levels, impairment of CCL19-driven CTL transmigration was observed at clinically relevant serum concentrations, suggesting distinct concentration thresholds for its effects on efflux activity versus migratory behavior.

These results suggest that JAK1/2 signaling is critical not only for cytokine responsiveness but also for chemokine-driven locomotion in human lymphocytes.

Moreover, the additive inhibitory effect of ruxolitinib on human dendritic cells and CD8^+^ T cells may underlie its therapeutic efficacy in conditions such as graft-versus-host disease (GVHD) and myeloproliferative disorders and may open new avenues for therapeutic applications targeting immune cell trafficking.

### 3.4. Clinical and Therapeutic Implications

The ability of ruxolitinib to modulate Pgp expression and function, suppress T cell activation markers, and inhibit chemokine-directed migration has significant therapeutic implications. These findings, together with others and our earlier observations [37], suggest that ruxolitinib can alter T cell differentiation pathways, may impair trafficking to inflammatory niches, and possibly influence immune exhaustion programs by reducing PD-1 expression. This multilayered modulation could enhance its utility in preventing aberrant T cell activation in autoimmune diseases, promoting tolerance in transplantation settings, and potentially reshaping immune memory in cancer immunotherapy. Although ruxolitinib promotes T cell memory differentiation, its inhibitory effect on bone marrow-directed migration suggests that, when combined with agents that facilitate homing to memory niches, it could be effectively incorporated into vaccination strategies to enhance long-term T cell persistence.

However, ruxolitinib’s interaction with Pgp also raises considerations for drug-drug interactions, as ruxolitinib may compete with other Pgp substrates for efflux and alter local drug concentrations within tissues.

### 3.5. Limitations and Future Directions

This study was conducted primarily using in vitro models, which, while informative, may not fully capture the complexity of immune interactions in vivo. Future studies should validate these findings in animal models or additional clinical samples, assess the long-term effects of ruxolitinib on memory T cell survival and function, and dissect the signaling pathways, JAK1/2 versus ROCK-dependent, that mediate the observed effects on migration and differentiation. Investigating how Pgp modulation differentially affects naïve, effector, and memory T cell subsets will also be crucial for optimizing therapeutic strategies.

## 4. Materials and Methods

### 4.1. Antibodies and Chemicals

The following fluorescently labeled monoclonal antibodies (mAbs) were applied. Mouse anti-human CD8 Allophycocyanin-Hilite7 (APC-H7) (Becton Dickinson, Budapest, Hungary). Mouse anti-human CD279 (PD-1) Allophycocyanin-Cyanine7 (APC-Cy7) (BioLegend, Biomedica Hungária Kft., Budapest, Hungary). The anti-human CD8 (OKT8) Pacific Orange and Pgp (15D3) Alexa Fluor 647 dye-labeled mouse monoclonal antibodies (mAbs) were produced in-house from hybridoma and mouse ascites. The isolation procedure was described earlier in detail [56].

### 4.2. Cell Lines and Generation of Cytotoxic T Lymphocytes

The P-glycoprotein (*MDR1/ABCB1*)-expressing cells, designated as NIH-3T3 MDR1, and their parental NIH-3T3 mouse fibroblast cell lines were obtained originally from the laboratory of Michel Gottesman, NIH, Bethesda, MD, USA. NIH-3T3 MDR1 cells are human P-glycoprotein knock-in cells, stably transfected into NIH-3T3 cells [57]. The JY cell line is a human Epstein–Barr virus-transformed B-lymphoblastoid cell line that expresses a high density of MHC class I A2 and class II DR on the surface plasma membrane [36,37]. Cell lines were maintained in RPMI 1640 medium (Sigma-Aldrich, Merck Life Science Kft., Budapest, Hungary) supplemented with sodium bicarbonate, glucose, pyruvate, MEM non-essential amino acids, GlutaMAX, gentamycin, or ampicillin, and 10% heat-inactivated FCS (SEBAC, Aidenbach, Germany) (complete medium) in 5% CO_2_ at 37 °C.

Human cytotoxic T lymphocytes (CTLs) were generated from peripheral blood lymphocytes (PBLs) using a previously established method with minor modifications, as described in prior studies [36,37,58,59]. The functionality, cytotoxic activity, and MHC class I-restricted antigen specificity of the resulting CTLs have been extensively validated in those works. Cells were collected, validated, and cryopreserved at the end of the expansion phase. Thawed CTLs were reused as needed for downstream experiments.

### 4.3. Measurement of P-Glycoprotein and CD8 Protein on Cell Surfaces

In this procedure, T cells were initially washed with PBS by centrifugation at 1200× *g* rpm for 5 min. After the washing step, the cells were counted using a hemocytometer and adjusted to a concentration of 5 million cells per mL. Subsequently, 250,000 cells were plated in a 96-well format. To facilitate the detection of P-glycoprotein and CD8, we added 10 µg/mL of Alexa647-15D3 anti-Pgp antibody and 10 µg/mL of Alexa488-OKT8 anti-CD8 antibody to the cells. The suspension was gently mixed and allowed to incubate on ice for 45 min. After incubation, the cells were washed twice with ice-cold glucose PBS to remove unbound antibodies. The cells were then resuspended in ice-cold glucose PBS containing 2 µM Hoechst dye. Pgp intensity measurements were subsequently performed using an ACEA Novocyte 3000 VBR flow cytometer (Accela, Budapest, Hungary).

### 4.4. Real-Time Quantitative PCR

Total RNA was isolated with TRIzolate Reagent (UD GenoMed, Debrecen, Hungary) and reverse transcribed to cDNA using a High-Capacity cDNA Reverse Transcription kit (Applied Biosystems, Budapest, Hungary) according to the manufacturer’s protocol. The amount of mRNA was determined by real-time q-PCR using TaqMan probes: Hs00184500_m1 *ABCB1* [60], Hs00184500_m1 *ACTB*, Mm02619580_g1 *Actb*, Hs02786624_g1 *GAPDH,* and Mm99999915_g1 *Gapdh*. Real-time monitoring was performed using a LightCycler 480 Instrument II (Roche, Budapest, Hungary). Gene expression normalization was conducted using both human and mouse *β-actin* and *GAPDH* reference genes, selected based on housekeeping gene stability analysis using the RefFinder tool (https://www.ciidirsinaloa.com.mx/RefFinder-master/, accessed on 24 June 2025). Gene expression was determined by the delta-delta cycle threshold (ΔΔC_T_) method.

### 4.5. Transport Activity Factor Assessment via Calcein Accumulation Assay

The transport activity factor was evaluated using adherent NIH-MDR1 and NIH-3T3 cell lines maintained at 80–90% confluence. The cells were thoroughly washed twice with 1x PBS, followed by trypsinization. The resulting cell suspension was counted using a hemocytometer and resuspended in 10 mL of 8 mM glucose PBS containing 1% FBS. This suspension was centrifuged at 1200 rpm for 5 min. After centrifugation, the supernatant was carefully discarded, and the pellet was resuspended in 1x 8 mM glucose-PBS to achieve a cell concentration of 20 × 10^6^ cells/mL. Similarly, suspension cells were washed twice with 1x PBS, reaching a final concentration of 20 × 10^6^ cells/mL using 1x glucose-PBS. The cells were then equally distributed into sorter tubes, with each tube containing 0.5 × 10^6^ cells. P-glycoprotein inhibitors, zosuquidar (1 µM), cyclosporine A (10 µM), and ruxolitinib (400 nM), were introduced to the samples. Calcein-AM, recognized as an ABCB1 substrate, was added at a final concentration of 100 nM. The treated cells were incubated under controlled conditions of 37 °C and 5% CO_2_ for a duration of 30 min. Subsequent to the incubation period, Alexa-546-15D3 and Alexa647-OKT8 antibodies were introduced into the samples. After an additional 30 min, the cells were washed with 3 mL of ice-cold 8 mM glucose-PBS containing 1% FBS to eliminate any excess calcein. The cells were then suspended in 0.5 mL of 2 µM Hoechst (a nuclear dye) and maintained on ice in the dark until measurements were conducted. Flow cytometric analysis was performed utilizing the BD Biosciences FACS Aria III (Budapest, Hungary). The transport activity factor of the P-glycoprotein pump was determined by analyzing the difference in dye accumulation within the cells in the presence versus the absence of the inhibitors.

TAF calculation: TAF = (MFI(inh) − MFI(0))/MFI(inh)(1)

MFI(inh): Mean Fluorescence Intensity with inhibitor; MFI(0): Mean Fluorescence Intensity without inhibitor. The TAF ranges from a maximum value of 1 to a minimum value of 0.0 [2,61].

### 4.6. Assessing ABC Transporter Activity Using Calcein-AM Retention and Flow Cytometry

The functional activity of ABC transporters was assessed through the inhibitory effects of specific inhibitors; to achieve this, a calcein accumulation assay was employed [2,38,39,40]. The ABCB1 inhibitor zosuquidar and the JAK1/2 kinase inhibitor ruxolitinib were analyzed. Drugs were serially diluted across 12 wells of a U-bottom 96-well plate, ranging from high to low concentrations. Calcein-AM, an ABC substrate, was added to achieve final concentrations of 5 nM for NIH-MDR cells and 1 nM for CTLs. Cells were evenly distributed in wells, with and without calcein-AM, at a density of 5 × 10^5^ cells per well. Following this, anti-CD8 Alexa647-OKT8 antibodies were added, and the treated cells were incubated at 37 °C in a controlled environment with 5% CO_2_ for 30 min. After incubation, the cells were washed twice with 200 µL of ice-cold glucose-PBS containing 1% FBS to remove excess calcein-AM. This was performed at 1200 rpm for 5 min. The treated cells were then suspended in 150 µL of 20 µg/mL Hoechst (nuclear dye) and maintained on ice in dark conditions. The intensity of calcein was subsequently measured using the ACEA Novocyte 3000 VBR flow cytometer.

IC_50_ values were determined using the pharmacological analysis toolkit in GraphPad Prism 8. Transport activity factor (TAF) values were first normalized to the maximum response, based on the corresponding base-10 logarithm of the molar inhibitor concentrations. The dose-response curves were then fitted using the “log (inhibitor) vs. normalized response” model.

### 4.7. Experimental Setup of T Cell Activation and Treatment

TCR activation of cells was achieved by coating wells of a 24-well plate with 2 µg/mL antibody of the CD3/CD28 Dynabeads and incubating overnight at 4 °C. Then, excess antibody was removed with a PBS wash, and one million cells from JY-exposed primed and JY-non-exposed unprimed cells were added separately to individual wells (TCR-activated cells). Ruxolitinib (MedChem Express, Budapest, Hungary) was added to wells separately containing CD3/CD28 (activated and RUX-treated cells). RUX was added to the wells that included only the cells (RUX-only treated cells), and untreated/unstimulated cells (control cells) were used. The activations and treatments were performed for 72 h (Table 1).

After the incubation period, the cells were counted, and a sample of 250,000 cells from each treatment condition was labeled with specific antibodies: anti-PD1 (APC-tagged), anti-Pgp (Alexa488-tagged UIC2 mAb combined with the Pgp inhibitor zosuquidar), and anti-CD8^+^ (Pacific Orange-tagged OKT8 mAb) in a 96-well U-bottom plate. The labeling procedure was conducted on ice for 45 min in the dark. Following this, the cells were washed once with PBS, resuspended in glucose PBS containing Hoechst, and subsequently analyzed using flow cytometry.

### 4.8. Preparation of Membrane Suspension for ATPase Activity Measurements

To measure Pgp-specific ATPase activity, we applied raw membrane samples prepared from NIH-3T3 mouse fibroblast cells expressing human Pgp at a high level. Membrane samples were prepared by using the methodology by Sarkadi et al. [44] with minor modifications previously described in [45,46]. Cells were harvested by scraping them into ice-cold PBS and washed twice at 300× *g* for 5 min. After this point, the whole procedure was performed in the presence of protease inhibitor cocktail (Sigma-Aldrich, Budapest, Hungary) and 0.5 mM phenylmethylsulphonyl fluoride (PMSF) at 4 °C to prevent the degradation of membrane proteins. Cells (120–150 × 10^6^) were homogenized in ice-cold TMEP buffer (50 mM Tris-HCl (pH 7.0), 50 mM mannitol, 2 mM EGTA, 2 mM DTT) using a glass-Teflon tissue homogenizer (Sigma-Aldrich, Budapest, Hungary). The homogenized cells were then centrifuged at 465× *g* for 10 min at 4 °C to sediment intact cells and nuclear debris. Then the supernatant containing cellular membranes was centrifuged at 28,000× *g* for 1 h at 4 °C. After centrifugation, the membrane pellet was resuspended in 1–2 mL TMEP buffer and stored at −80 °C until further use. The protein concentration of the membrane suspension was measured by using the Lowry method [43].

### 4.9. ATPase Activity Measurements

The Pgp-specific ATPase activity was determined by measuring the amount of inorganic phosphate liberated as a result of ATP hydrolysis. Other membrane-bound ATPases significantly contributing to cellular ATP consumption were blocked by Na-azide (for the F0F1 complex), ouabain (for Na^+^/K^+^ ATPase), and EGTA (for Ca^+2^ ATPases) [44]. Membrane samples (5 μg membrane protein/sample) were pre-incubated with the tested drugs (1–100 µM ruxolitinib, 10 µM zosuquidar, or 40 µM verapamil) in 60 μL ATPase assay premix (50 mM MOPS, 65 mM KCl, 6.5 mM NaN3, 2.6 mM DTT, 1.28 mM ouabain, 0.65 mM EGTA, pH = 7.0) in the presence or absence of 100 μM Na3VO4 (vanadate) for 10 min at 37 °C. Then, the ATPase reaction was initiated by adding 3.5 mM MgATP to each sample. After 25 min incubation at 37 °C, the ATPase reaction was stopped by 40 μL 5% SDS, and then the samples were further incubated with 105 μL color reagent [42] at room temperature for 30 min. Absorbances of the samples were measured at 700 nm using a BioTek Synergy HT plate reader (BioTek Instruments, Winooski, VT, USA), and the amount of released Pi was calculated by preparing a calibration curve using samples with known Pi concentration. Since vanadate is a specific inhibitor of ATP hydrolysis by ABC transporters, the Pgp-specific ATPase activity is determined by calculating the difference between the vanadate-untreated and vanadate-treated sample pairs [44].

### 4.10. Flow Cytometry

Calcein intensity was systematically measured in NIH-3T3 MDR1, NIH-3T3, and CTLs, alongside the expression levels of P-glycoprotein and CD8 in CTLs. Cellular surface staining of lymphocytes was performed using the mAbs detailed above. Briefly, cells were incubated with mAbs for 15 min at room temperature, followed by washing with PBS at 1600 RPM for 5 min. Later, cells were suspended in 400 µL of PBS for acquisition. These evaluations utilized the ACEA Novocyte 3000 VBR and BD Biosciences FACS Aria III flow cytometers. The resulting data from the FACS and flow cytometry analyses were processed using FCS Express version 6.0 Flow Research Edition (De Novo Software, Pasadena, CA, USA).

### 4.11. CTL Transmigration Assay Across HUVEC Monolayers

*HUVEC isolation and culture.* Human umbilical vein endothelial cells were isolated as previously described by Palatka et al. [62] and stored frozen until use. For experiments, cells were thawed and cultured in M199 medium (Biosera, Cholet, France) supplemented with 10% heat-inactivated fetal bovine serum (Thermo Fisher Scientific, Budapest, Hungary), 100 U/mL penicillin, 100 μg/mL streptomycin, 2.5 μg/mL amphotericin B (Biosera, Cholet, France), 2 mM glutamine (Biosera, Cholet, France), and 10% Endothelial Cell Growth Medium-2 (Lonza, Budapest, Hungary). Cells were maintained at 37 °C with 5% CO_2_ (Eppendorf, Wien, Austria) in gelatin-coated T75 flasks until confluence, then detached with trypsin-EDTA and washed with sterile PBS.

*HUVEC monolayer preparation.* For transmigration assays, 1.5 × 10^5^ HUVECs were seeded onto 24-well transwell inserts (5 μm pore size; CellQART, SABEU GmbH & Co., Radeberg, Germany) pre-coated with 0.2% gelatin. The medium was refreshed every three days. Monolayer integrity was confirmed by measuring transendothelial electrical resistance (TEER) and by mosaic imaging of Calcein Green-AM-stained live HUVECs using a laser-scanning cytometer (iCys, CompuCyte, Boston, MA, USA) after cyclosporin A treatment. Plateaued resistance by day 5 indicated confluency.

*CTL processing and transmigration.* Human cytotoxic T lymphocytes were labeled with Calcein Green-AM and Hoechst 33342 (Thermo Fisher Scientific, Budapest, Hungary). In some experiments, HUVEC monolayers were counterstained with Calcein Red-AM (Invitrogen, Budapest, Hungary). CTLs were pretreated overnight with 100 nM ruxolitinib or with varying concentrations (0.04, 0.4, and 4 μg/mL) in complete medium.

For transmigration, 3 × 10^5^ labeled CTLs in 340 μL RPMI 1640 medium supplemented with 0.5% fatty acid-free BSA (Sigma-Aldrich, Budapest, Hungary) were added to the upper chambers. Lower wells contained 360 μL RPMI with 500 ng/mL CCL19 chemokine (Sigma-Aldrich, Budapest, Hungary) as a chemoattractant. Cells were allowed to migrate for 4 h at 37 °C.

Cells from the upper and lower compartments, as well as from the underside of the inserts (detached by trypsinization), were collected. Calcein Green and Hoechst-positive viable cells were quantified using an ACEA NovoCyte flow cytometer (Accela, Budapest, Hungary) and analyzed with FCS Express version 6.0 (De Novo Software, Pasadena, CA, USA).

All conditions were tested in triplicate, and experiments were independently repeated at least three times.

### 4.12. Statistical Analysis

Kolmogorov–Smirnov and Shapiro–Wilk normality tests were used to test the normality of data distribution. For non-normally distributed, nonparametric variables, the Mann–Whitney test and normally distributed parametric variables, Student’s t-test were used to calculate the significant differences.

One-way ANOVA with Tukey’s post hoc test for multiple comparisons was employed for internal comparison with the level of significance set at *p* < 0.05. Statistical analysis was performed using GraphPad Prism v9.0 (GraphPad Software, Inc., San Diego, CA, USA).

### 4.13. Ethics Approval

Blood samples were obtained with the written consent of voluntary healthy donors through individual donations at the Regional Blood Center of the Hungarian National Blood Transfusion Service (Debrecen, Hungary). This collection was conducted under the written approval (OVSzK 3572-2/2015/5200) of the Director of the National Blood Transfusion Service and the Regional and Institutional Ethics Committee of the University of Debrecen, Medical and Health Science Center (Hungary).

## 5. Conclusions

In conclusion, our findings reveal that ruxolitinib exerts multifaceted effects on human cytotoxic T lymphocytes, modulating P-glycoprotein expression and activity, suppressing activation-associated surface markers, and impairing chemokine-driven endothelial transmigration. These effects suggest that ruxolitinib may influence T cell differentiation trajectories, migratory behaviors, and functional adaptation to tissue microenvironments.

Notably, while inhibition of Pgp efflux function and ATPase modulation required concentrations higher (in the range of 10–100 µM) than typical therapeutic levels (1–3 µM plasma peak concentrations), the impairment of CCL19-driven CTL transmigration and mRNA alterations were observed at clinically relevant serum concentrations (in the range of 0.1–5 µM), suggesting distinct concentration thresholds for its effects on efflux activity versus migratory and mRNA induction behaviors.

Given its ability to impair lymphocyte trafficking at clinically relevant concentrations, ruxolitinib may not only suppress pathogenic immune activation, as in graft-versus-host disease (GVHD), but could also be strategically leveraged to influence memory T cell localization during vaccination or immunotherapeutic interventions.

Understanding how JAK inhibition alters immune cell dynamics at both molecular and functional levels may inform the development of optimized therapeutic strategies in transplantation, autoimmune diseases, and cancer immunotherapy. Future studies will be necessary to elucidate further how ruxolitinib influences the fate, persistence, and tissue-specific homing of memory T cells in vivo.

## Figures and Tables

**Table 1:** CD3/CD28 activation of human primary peripheral blood lymphocytes over 72 h.

Activation Status	Untreated	Drug Treated
No	Human PBLs	Human PBLs +RUX
Yes	Human PBLs +CD3/CD28 beads	Human PBLs +CD3/CD28 beads +RUX

Each well contained two million PBLs treated with the JAK kinase inhibitor ruxolitinib (RUX) at a concentration of 100 nM. Cells were activated using anti-CD3/anti-CD28 antibody-coated beads at 2 µg/mL, with or without RUX treatment, as outlined in the experimental setup described above.

## Data Availability

Data is contained within the article.
